# Polysaccharides from astragali radix restore chemical-induced blood vessel loss in zebrafish

**DOI:** 10.1186/2045-824X-4-2

**Published:** 2012-02-23

**Authors:** Guang Hu, Gail B Mahady, Shang Li, Maggie Pui Man Hoi, You-Hua Wang, Simon Ming Yuen Lee

**Affiliations:** 1 State Key Laboratory of Quality Research in Chinese Medicine (University of Macau), Avenue Padre Tomás Pereira S.J., Macao SAR, China; 2 Institute of Chinese Medical Sciences, University of Macau, Avenue Padre Tomás Pereira S.J., Taipa, Macao SAR, China; 3 Departments of Pharmacy Practice and Pan-American Health Organization/World Health Organization Collaborating Centre for Traditional Medicine, College of Pharmacy, University of Illinois at Chicago, Chicago, USA; 4 Longhua Hospital, Shanghai University of Traditional Chinese Medicine, Shanghai, China

**Keywords:** Angiogenesis, Astragali Radix, Polysaccharide, Ultrafiltration, Zebrafish

## Abstract

**Background:**

Astragali Radix has been used widely for the treatment of cardiovascular and cerebrovascular diseases, and to enhance endurance and stamina in traditional Chinese medicine (TCM) for over 2000 years. The polysaccharide constituents of Astragali Radix (ARP) are considered as one of the major constituents contributing to the multiple pharmacological effects of this medicinal plant. The purpose of the study is to evaluate the vascular regenerative activities of ARPs in a chemically-induced blood vessel loss model in zebrafish.

**Methods:**

Blood vessel loss was induced in both Tg(fli-1a:EGFP)y1 and Tg(fli-1a:nEGFP)y7 embryos by administration of 300 nM VEGFR tyrosine kinase inhibitor II (VRI) for 3 h at 24 hpf (hour post-fertilization). Then, the blood vessel damaged zebrafish were treated with ARPs for 21 h and 45 h after VRI withdrawal. Morphological changes in intersegmental vessels (ISVs) of zebrafish larvae were observed under the fluorescence microscope and measured quantitatively. The rescue effect of ARPs in the zebrafish models was validated by measuring the relative mRNA expressions of Kdrl, Kdr and Flt-1 using real-time PCR.

**Results:**

Two polysaccharide fractions, P4 (50000 D < molecular weight & diameter < 0.1 μm) and P5 (molecular diameter > 0.1 μm), isolated from Astragali Radix by ultrafiltration, produced a significant and dose-dependent recovery in VRI-induced blood vessel loss in zebrafish. Furthermore, the down-regulation of Flk-1 and Flt-1 mRNA expression induced by VRI was reversed by treatment with P4.

**Conclusion:**

The present study demonstrates that P4 isolated from Astragali Radix reduces VRI-induced blood vessel loss in zebrafish. These findings support the hypothesis that polysaccharides are one of the active constituents in Astragali Radix, contributing to its beneficial effect on treatment of diseases associated with a deficiency in angiogenesis.

## Background

Angiogenesis plays an important role in a wide range of physiological processes, such as wound healing and fetal development. However, many diseases such as cancer, chronic inflammatory disease, diabetic retinopathy, macular degeneration and cardiovascular disorders are associated with dysregulation of angiogenesis, in which blood vessel formation is either excessive or insufficient. Improvement of endothelial cell function and the enhancement of angiogenesis after critical cardiac and skeletal muscle ischemia is critical, as neovascularization of ischemic tissues may be sufficient to preserve tissue integrity and/or function, and thus is therapeutic. Polysaccharides are naturally occurring polymeric carbohydrate structures formed of repeating units of mono- or di-saccharides joined together by glycosidic bonds. This group of natural compounds are present in many traditional Chinese herbs and are reported to have both pro-angiogenic [1,2] and anti-angiogenic [3-6] activities. Our previous discovery of a pro-angiogenic herb called *Angelica sinesis *by zebrafish assay leading to development of a wound healing formulation for diabetic foot ulcer patients [7,8].

Astragali Radix, the dried root of *Astragalus membranaceus *(Fisch) Bge. or *Astragalus mongholicus *Bge. (Fabaceae), has been used in traditional Chinese medicine (TCM) for centuries to enhance the immune system, increase stamina and endurance, and to treat cerebrovascular and cardiovascular diseases [9]. In China, the herb is commonly known as "Huangqi", and was first recorded in Shen Nong's Materia Medica about two thousand years ago. The primary constituents of Astragali Radix include polysaccharides, triterpene saponins, flavonoids, amino acids and trace elements [10,11]. Clinically, Astragali Radix is used as either a single herb or in a TCM formula in combination with other herbal medicines. As a single herb, it stimulates the formation of capillaries in the chick embryo chorioallantoic membrane, and induces the proliferation of human umbilical vein endothelial cells (HUVEC) [12,13].

In our previous study, an extract of Astragali Radix containing flavonoids, saponins and polysaccharides stimulated angiogenesis involving the VEGF-KDR/Flk and PI3K-Akt-eNOS pathways [14]. Calycosin, one of the major isoflavones in Astragali Radix, was found to promote angiogenesis in normal zebrafish [15], whereas astragaloside IV reduced chemically-induced blood vessel loss [16]. However, there are no systematic and in-depth studies investigating the angiogenesis activities of fractionated polysaccharides from Astragali Radix (ARPs).

New opportunities for *in vivo *natural product discovery have arisen through the recent emergence of zebrafish as an effective model system for the identification of disease-relevant genes and bioactive small molecules [17]. The primary advantages of zebrafish for drug discovery include their high genetic, physiologic, and pharmacologic similarity with humans [17,18]. In particular, *in vivo *screening of the angiogenic effects of ARP in zebrafish provides a more physiologically relevant result compared to *in vitro *screening because polysaccharides are often subjected to modification by the gastrointestinal tract and drug metabolism systems *in vivo*. Also, our recent studies suggest a high similarity of phase I and phase II drug metabolism systems between zebrafish and mammals [19,20]. In the present study, we have investigated the vascular effects of fractionated ARPs based on molecular size in zebrafish angiogenesis assays.

## Materials and methods

### Ethics statement

All animal experiments were conducted according to the ethical guidelines of ICMS, University of Macau and the protocol was approved by ICMS, University of Macau prior to the initiation of the experiments.

### Chemicals and reagents

Dimethyl sulfoxide (DMSO) was purchased from Sigma, St. Louis, USA. VEGFR tyrosine kinase inhibitor II (VTKI, VRI) was purchased from Calbiochem Company/EMD Chemicals Inc (Cat. No. 676481) and was dissolved in DMSO to form a 1 mg/mL solution. Purified water was prepared with a Milli-Q purification system from Millipore (Milford, USA).

### Preparation and analysis of ARP fractions

The dry roots of Astragali Radix (3,000 g) were extracted twice with 30 L distilled water at 80°C for 2 hours, and then filtered to remove impurities. The aqueous extract was concentrated and applied to an HPD600 macroporous resin column for further separation. The column was eluted with 10 L of ethanol and the aqueous portion was collected by eluting with 12 L of water, and then precipitated by the addition of three volumes of 80% (v/v) ethanol, and the precipitate was left to stand overnight. The resultant precipitate was collected by centrifugation, and then dried under vacuum. This dried polysaccharide extract was re-dissolved in distilled water and treated with Sevag reagent (1:4 n-butanol:chloroform, v/v, 3000 mL × 5) to remove proteins. The deproteined polysaccharide fraction was further fractionated using ultrafiltration with a series of commercial ultrafiltration membranes (size 10 K, 30 K, 50 K and 0.1 μm, Millipore, USA), according to the molecular weight (MW) and diameter (DM) of the target polysaccharide fraction.

The total sugar content was determined by a modified phenol/sulfuric acid method [21] at each step, and the yields of each polysaccharide fraction were calculated. Furthermore, the number-average molecular weight (Mn) and the weight-average molecular weight (Mw) of the active ARP fractions with higher efficacy and potency (P4) were determined by gel permeation chromatography (GPC). The uronic acid content of P4 was also determined using the hydroxydiphenyl method as previously described [22]. The monosaccharide units of P4 were analyzed by gas chromatography (GC), and several types of monosaccharides were used as reference standards.

### Maintenance of zebrafish and its embryos

The zebrafish strains, including Tg(fli-1a:EGFP)y1 and Tg(fli-1a:nEGFP)y7, were maintained as previously described (Westerfield et al. 1995).

### Zebrafish embryo collection and drug treatment

Zebrafish embryos were generated by natural pair-wise mating (3-12 months old) and were raised at 28.5°C in embryo medium. Healthy, hatched zebrafish were picked out at 24 hour post-fertilization (hpf) and distributed into a 12-well microplate (15 embryos per well). They were pretreated with 300 nM tyrosine kinase inhibitor II, one of the VEGF receptor inhibitors (VRI), for 3 h. After that, the VRI was washed out and replaced with either 0.1% DMSO (v/v) embryo medium, or increasing concentrations of each of the five ARP fractions for 45 h. The embryos receiving embryo medium only served as a vehicle control. The media of all treatment groups were refreshed every 24 h. Each experiment was repeated at least three times.

### Morphological observation of zebrafish

At 48 hpf and 72 hpf, zebrafish were removed from microplates and observed for viability and gross morphological changes under a fluorescence microscope (Olympus IX81 Motorized Inverted Microscope, Japan) equipped with a digital camera (DP controller, Soft Imaging System, Olympus). Images were analyzed with Axiovision 4.2 and Adobe Photoshop 7.0.

### Assessment of vascular changes

24 hpf Tg(fli-1a:EGFP)y1 zebrafish embryos were pretreated with VRI for 3 h. Then after treatments of ARPs for 21 h and 45 h, intersegmental vessels (ISVs) were assessed for pro-angiogenesis activity of ARPs. In the vehicle control group, ISVs can be seen sprouting and elongating dorsally up from the dorsal aorta (DA), and the posterior cardinal vein (PCV) below, to form a right and left pair of dorsal longitudinal anastamotic vessels (DLAVs) by 1.5 dpf [23]. In the vehicle control group, ISVs observed sprouting and elongating from DA (and CA) or PCV to DLAV were defined as intactness. In VRI treatment groups (including the VRI-only treatment group and the ARP treatment groups), some of the ISVs observed sprouting from DA or PCV, but not forming a complete ISV, were defined as a defective. The integrity of twenty ISVs near the anus (ten ISVs in front of the anus and ten ISVs after the anus) in each individual zebrafish was measured. The percentage recovery was calculated as the total length of the selected ISVs in the treatment group over the total length of the selected ISVs in the vehicle control group. For example: A 100% percentage recovery indicated completed recovery of ISVs in the treatment group the same as that in the vehicle control group, whereas the VRI-only treatment group showing complete inhibition of ISVs was set as 0% percentage recovery. Twenty ISVs near the anus (10 ISVs in front of the anus and 10 ISVs after the anus) in each embryo were measured for total length. At least 10 larvae per group, over a series of 3 independent experiments, were evaluated. Percentage recovery is defined as the total length of the selected ISVs in the treatment group over the total length of the selected ISVs in the vehicle control group. In addition, the total number of endothelial cells in the ISVs of Tg(fli-1a:nEGFP)y7 zebrafish larvae were assessed by counting directly the number of nuclei of GFP-positive endothelial cells present in the twenty selected ISVs. Each green light point represents one endothelial cell (GFP+). Data are presented as the mean ± SD (n = 3),* *P *< 0.05, # *P *< 0.001.

### Total RNA extraction, reverse transcription, and real-time PCR

The zebrafish embryos were treated at 24 hpf as described above. At 48 hpf, total RNA was extracted from 30 zebrafish embryos of each treatment group, using the RNeasy Mini Kit (Qiagen, USA) in accordance with the manufacturer's instructions. RNA was reverse transcribed to single-strand cDNA using SuperScriptTM III First-Strand Synthesis System for RT-PCR (InvitrogenTM, USA), followed by real-time PCR using the TaqManH Universal PCR Master Mix, and 250 nM custom TaqMan primers for zebrafish Kdrl, Kdr and Flt1 (Applied Biosystems, USA) in the ABI 7500 Real-Time PCR System (Applied Biosystems). The expression of Kdrl, Kdr and Flt1 was normalized to the amount of bactin1, using the relative quantification method described by the manufacturer.

The zebrafish bactin1 primers were 5'-CAAGATTCCATACCCAGGAAGGA- 3' (F) and 5'-CAAGATTCCATACCCAGGAAGGA- 3' (R) (Applied Biosystems, USA).

The zebrafish Kdrl (Flk1A) primers were 5'- GACCATAAAACAAGTGAGGCAGAAG- 3' (F) and 5'- CTCCTGGTTTGACAGAGCGATA- 3' (R) (Applied Biosystems, USA).

The zebrafish Kdr (Flk1B) primers were 5'- CAAGTAACTCGTTTTCTCAACCTAAGC- 3' (F) and 5'-GGTCTGCTACACAACGCATTATAAC- 3' (R) (Applied Biosystems, USA).

The zebrafish FLT1 primers were 5'-AACTCACAGACCAGTGAACAAGATC- 3' (F) and 5'-GCCCTGTAACGTGTGCACTAAA- 3' (R) (Applied Biosystems, USA).

## Results

### Preparation of ARP fractions

Five ARP fractions were prepared by extraction, isolation, deproteination and ultrafiltration: P1 (MW < 10,000 D, 15 g), P2 (10,000 D < MW < 30,000 D, 25.2 g), P3 (30,000 D < MW < 50,000 D, 55.3 g), P4 (50,000 D < MW & DM < 0.1 μm, 75.5 g) and P5 (DM > 0.1 μm, 156 g). The total sugar content of each polysaccharide fraction was: P1, 35.9%; P2, 36.2%; P3, 42.4%; P4, 42.9%; P5, 51.1%. The yield of each fraction was: P1, 0.5%; P2, 0.84%; P3, 1.84%; P4, 2.52%; P5, 5.12% (Table 1). The number-average molecular weight (Mn) and weight-average molecular weight (Mw) of P4 was 6.46 × 10^5 ^D and 1.61 × 106 D (see Additional File 1). The uronic acid content of P4 was determined to be 26.9%. Results from gas chromatography (GC) revealed that the monosaccharides of P4 consisted of D-glucose, D-galactose and D-glucuronic acid (see Additional File 2).

**Table 1:** Description of ARPs extracted from Radix Astragali

	P1	P2	P3	P4	P5
**molecular weight (D) and diameter range (μm)**	**molecular weight < 10000**	**10000 < molecular weight < 30000**	**30000 < molecular weight < 50000**	**50000 < molecular weight &diameter < 0.1**	**diameter > 0.1**

total sugar content (%)	35.9	36.2	42.4	42.9	51.1

yield (%)	0.5	0.8	1.8	2.5	5.1

maximum non-toxic dosage (μg/ml)	300.0	100.0	300.0	100.0	300.0

### ARP fractions rescue VRI-induced blood vessel loss in zebrafish

**Figure 1:**
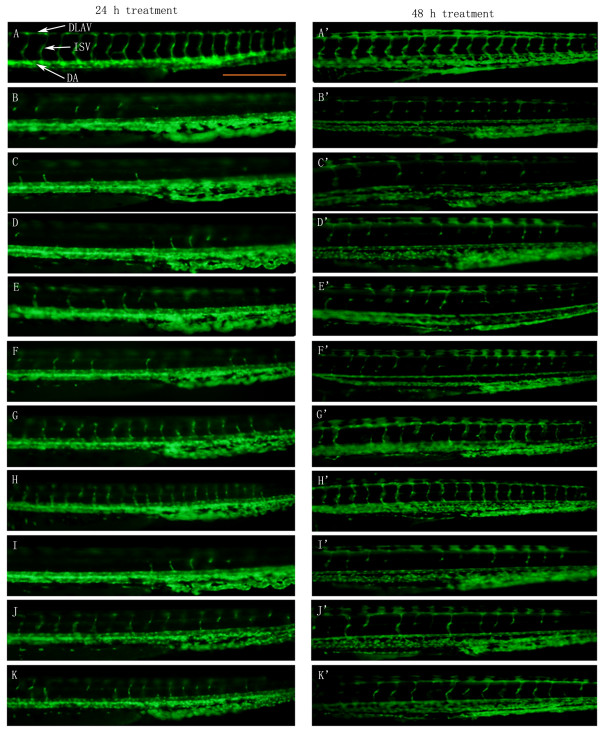
**The effect of ARPs on VRI-induced blood vessel loss in Tg(fli-1a:EGFP)y1 zebrafish (result of morphological observation)**. White arrows indicate DLAV, ISV and DA of zebrafish. Scale bar = 500 μm. (A-A') Control group: 24 hpf embryos were treated with 0.1% DMSO for 24 h and 48 h. 24 hpf embryos were treated with VRI (300 nM) at for 3 h. After that, the VRI was washed out and replaced with 0.1% DMSO (v/v) embryo medium (B-B') or 300 μg/ml P1 (C-C'), 100 μg/ml P2 (D-D'), 300 μg/ml P3 (E-E'), 10 μg/ml (F-F'), 30 μg/ml (G-G') and 100 μg/ml (H-H') P4, 30 μg/ml (I-I'), 100 μg/ml (J-J') and 300 μg/ml (K-K') P5 for 24 h.

VEGFR tyrosine kinase inhibitor II (VRI), a pyridinyl-anthranilamide compound that displays antiangiogenic properties, strongly inhibits the kinase activities of both VEGF receptor 1 and 2. The 27 hpf zebrafish embryos pre-treated with 300 nM of VRI for 3 h were then washed and placed in Milli Q water and incubated for another 45 h. Treatment of the zebrafish with VRI induces significant blood vessel loss in ISV (intersegmental vessels) and DLAV (dorsal longitudinal anastomotic vessels) (Figure 1A-A' and Figure 1B-B'). After incubating the VRI-pretreated embryos with P4 (30 and 100 μg/ml) (Figure 1F-H') and P5 (100 and 300 μg/ml) (Figure 1I-K'), respectively, for 21 h and 45 h, the VRI-induced blood vessel loss in the ISV and DLAV regions of the zebrafish was significantly reduced.

**Figure 2:**
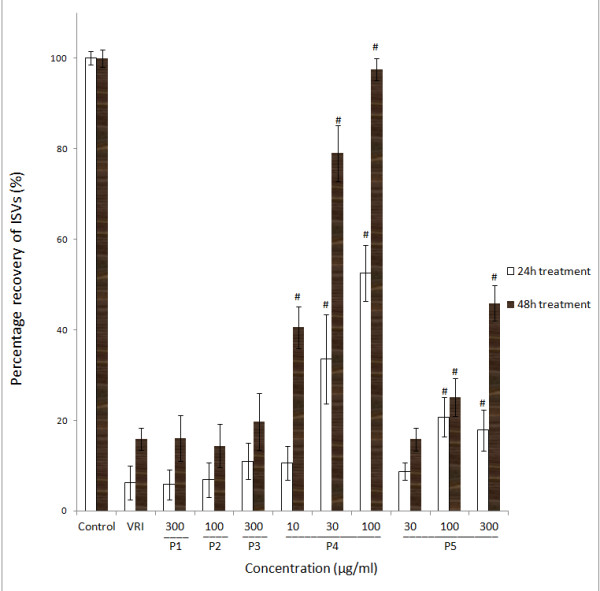
**Effects of ARPs on VRI-induced blood vessel loss in Tg(fli-1a:EGFP)y1 zebrafish (result of statistical analysis)**. Percentage recovery (the total length of the selected ISVs in the treatment group over the total length of the selected ISVs in the vehicle control group) of each ISV of Tg(fli-1a:EGFP)y1 zebrafish were calculated. Data are plotted as the mean ± SD, (n = 3), * *P *< 0.05, # *P *< 0.001 vs the VRI-only treatment group.

Quantitative analysis confirmed a significant (*P *< 0.05 and *P *< 0.001, respectively) dose-dependent effect of P4 and P5 on the percentage recovery (the total length of the selected ISVs in treatment group over the total length of the selected ISVs in the vehicle control group) of blood vessel loss in zebrafish as compared with the control group (Figure 2). Meanwhile, there was no significantly observable change in blood vessels in P1, P2 and P3 treatment groups at their maximum non-toxic concentrations of 300 μg/ml, 100 μg/ml and 300 μg/ml, respectively (Figure 1C-E'), compared with VRI-only treatment group.

**Figure 3:**
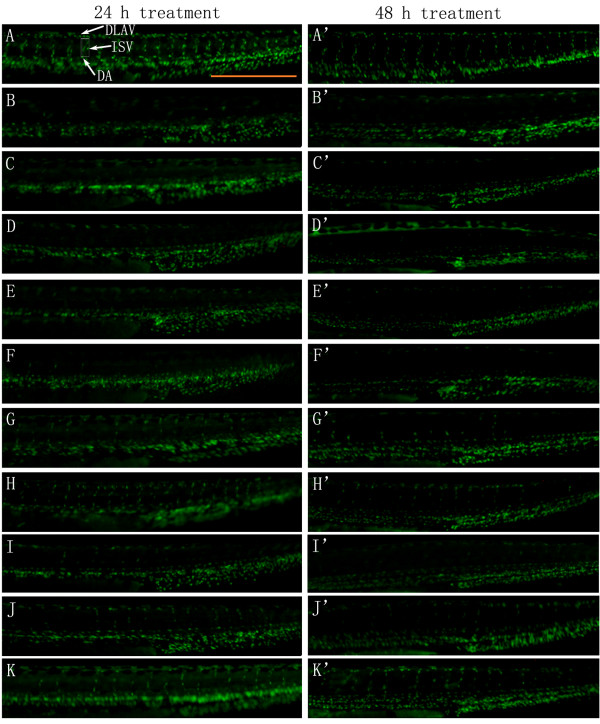
**The effect of ARPs on VRI-induced blood vessel loss in Tg(fli-1a:nEGFP)y7 zebrafish (result of morphological observation)**. White arrows indicate DLAV, ISV and DA of zebrafish. Scale bar = 500 μm. (A-A') Control: embryos treated with 0.1% DMSO at 24 hpf for 24 h and 48 h. 24 hpf embryos treated with VRI (300 nM) for 3 h. After that, the VRI was washed out and replaced with 0.1% DMSO (v/v) embryo medium (B-B') or 300 μg/ml P1 (C-C'), 100 μg/ml P2 (D-D'), 300 μg/ml P3 (E-E'), 10 μg/ml (F-F'), 30 μg/ml (G-G') and 100 μg/ml (H-H') P4, 30 μg/ml (I-I'), 100 μg/ml (J-J') and 300 μg/ml (K-K') P5 for 24 h.

**Figure 4:**
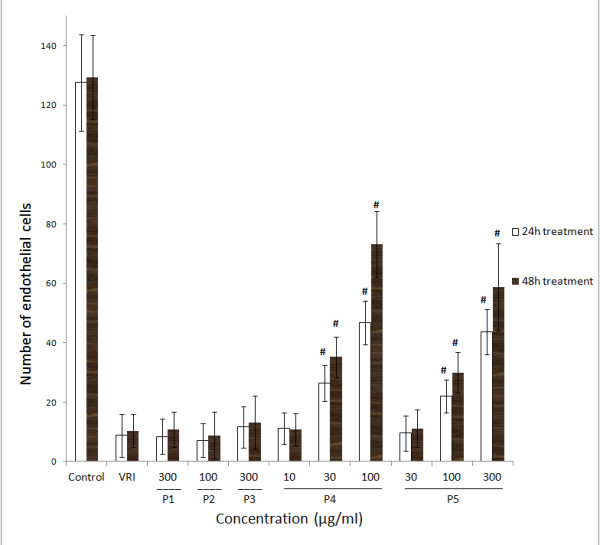
**Effects of ARPs on VRI-induced blood vessel loss in Tg(fli-1a:nEGFP)y7 zebrafish (result of statistical analysis)**. Numbers of endothelial cells in ISVs of Tg(fli-1a:nEGFP)y7 zebrafish embryos were assessed by direct counting of the total number of green light points within the twenty selected ISVs. Each green light point represents one endothelial cell (GFP+). Data are plotted as the mean ± SD, (n = 3), * *P *< 0.05, # *P *< 0.001 vs the VRI-only treatment group.

Moreover, Tg(fli-1a:nEGFP)y7 zebrafish were used to confirm the angiogenic effects of different fractions of ARP. Tg(fli-1a:nEGFP)y7 fish were engineered similarly to Tg(fli-1a:EGFP)y1, with the exception that the Tg(fli-1a:nEGFP)y7 harbor nuclear-localized GFP expression, permitting real time *in vivo *analysis of individual endothelial cells [24]. Compared with Tg(fli-1a:EGFP)y1 zebrafish that were used to evaluate the integrity of blood vessels, Tg(fli-1a:nEGFP)y7 zebrafish were used to measure the number of endothelial cells. The results showed that after incubating with 0.1% DMSO (v/v) embryo medium for 21 h and 45 h, the number of zebrafish larvae endothelial cells in the ISV and DLAV regions decreased dramatically following 0.3 μM VRI pre-treatment for 3 h (Figure 3B-B'), compared to the vehicle control (Figure 3A-A'). Similar to the results with Tg(fli-1a:EGFP)y1 (Figure 1), this VRI-induced blood vessel cell decrease was partially reduced after treatment with P4 (30 and 100 μg/ml) (Figure 3G-H') and P5 (100 and 300 μg/ml) (Figure 3I-K') for 21 h and 45 h. Quantitative analysis indicated that treatment of VRI-pretreated zebrafish with P4 (30 and 100 μg/ml) and P5 (100 and 300 μg/ml) significantly (*P *< 0.01 and *P *< 0.001) increased the number of endothelial cells (Figure 4) throughout the ISV region than the VRI-only treatment group. P1 (300 μg/ml), P2 (100 μg/ml) and P3 (300 μg/ml) did not show significant observable rescue effects on VRI-pretreated zebrafish larvae (Figure 3C-E'). Since P4 exhibited higher potency and efficacy than P5 on the recovery of blood vessel loss in both Tg(fli-1a:EGFP)y1 and Tg(fli-1a:nEGFP)y7 zebrafish lines, P4 was selected for further mechanistic studies.

### P4 reverses the VRI induced down regulation of flk-1 and flt-1 mRNA expression

In order to confirm the vascular phenotypic effects of P4 observed in Figure 1, 2, 3, 4, total RNAs from different groups of zebrafish pre-treated with VRI (0.3 μM) for 3 h, and then treated with different concentrations of P4 (10, 30 and 100 μg/ml) after VRI withdrawal, were isolated and reverse transcribed to cDNA, and relative mRNA expressions of Kdrl, Kdr and Flt-1 were determined, using real-time PCR. Vascular endothelial growth factor receptor 1 (VEGFR1) is a protein that in zebrafish is encoded by the FLT1 gene [25]. Its tyrosine protein kinase activity is important for the control of cell proliferation and differentiation. VEGFR2 (fetal liver kinase, also known as KDR and Flk-1) has a higher affinity for VEGF and is a major transducer of the VEGF signal in endothelial cells [26,27]. Figure 7 represents the gene expression levels (data expressed in a log_2 _scale) of three key VEGFRs in 48 hpf zebrafish larvae that were pre-treated with 0.3 μM VRI for 3 h and then treated with P4 (10, 30, and 100 μg/ml) for 21 h after VRI withdrawal. P4 caused a significant increase in mRNA expression of Kdrl (2.31-fold at 10 μg/ml, *P *< 0.001; 2.25-fold at 30 μg/ml, *P *< 0.001; 2.58-fold at 30 μg/ml, *P *< 0.001), Kdr (2.67-fold at 10 μg/ml, *P *< 0.001; 2.71-fold at 30 μg/ml, *P *< 0.001; 2.85-fold at 100 μg/ml, *P *< 0.001) and Flt1 (1.77-fold at 10 μg/ml, *P *< 0.001; 1.63-fold at 30 μg/ml, *P *< 0.001; 1.96-fold at 100 μg/ml, *P *< 0.001). Thus, these results suggest that P4 reversed the down-regulation of the expression of several key angiogenesis genes involved in VRI-induced blood vessel loss in zebrafish.

## Discussion

Astragali Radix has been used as a single herb, or in combination with other Chinese herbal medicines, for the treatment of cardiovascular diseases and to enhance stamina and endurance for thousands of years, which prompted us to investigate the vascular protective effects of ARPs from Astragali Radix. In the present study, ARPs were isolated by ultrafiltration according to different molecular weight ranges, and evaluating their pro-angiogenesis effects using the live zebrafish angiogenesis assays.

The results demonstrated that the Astragali Radix polysaccharide fraction P4 (ranging from MW > 50,000 D and DM < 0.1 μm) and P5 (range: DM > 0.1 μm) reduced VRI-induced blood vessel loss in zebrafish models. Both the percentage recovery and the number of endothelial cells of ISVs increased significantly following treatments with P4 and P5 in both the transgenic Tg(fli-1a:EGFP)y1 and Tg(fli-1a:nEGFP)y7 zebrafish models as compared with the VRI-only treatment group. These findings indicate that P4 and P5 possess pro-angiogenic activity. Compared to P5, the ARP fraction P4 exhibited higher potency and efficacy.

The results also demonstrated that P4-treatments induced phenotypic change in VRI-induced blood vessel loss in the zebrafish model that were well validated by recovery of the mRNA expressions of a few key gene makers selected from angiogenesis signaling pathways. VEGF, also known as vascular permeability factor (VPF), was originally described as a potent angiogenic factor as well as an essential growth factor for vascular endothelial cells [28]. The formation of new blood vessels is orchestrated by a variety of different proteins, including cell adhesion molecules, extracellular matric components and VEGFRs. Gene targeting experiments have provided insights into the functions of VEGFRs [29,30]. Although inactivation of each individual VEGFR can cause embryonic lethality at mid-gestation, they have different functionality [31,32]. VEGFR2 is the receptor that initiates the main signaling pathways activated by VEGF. While the main function of VEGFR1 appears to be in regulating the binding between VEGF and VEGFR2 [33]. In this investigation, the results of real-time PCR illustrate that different concentrations (10, 30 and 100 μg/ml) of P4 reversed VRI-induced down-regulation of the expression of Kdrl, Kdr and Flt1 in the zebrafish model. These data confirmed the predominant involvement of these angiogenesis-specific targets in P4's rescue effect at ISVs in VRI-induced blood vessel loss in zebrafish, and further supported the hypothesis that these clear phenotypic changes were the result of the recovery of angiogenesis deficiency.

Angiogenesis deficiencies are associated with numerous human cardiovascular and cerebrovascular diseases (*e.g.*, ischemic cardiac and cerebral problems). The work presented here shows that VRI-induced blood vessel loss in zebrafish *in vivo *mimics angiogenesis deficiencies associated with human disease conditions. Our *in vivo *data suggests that P4 exerted a rescue effect only in a damaged blood vessel disease model rather than in healthy zebrafish (data not shown). This strongly suggests that P4 may be a novel therapy for the restoration of angiogenesis deficiencies under pathophysiological conditions. In summary, these data suggest that P4 exerts its angiogenic effects by rescuing damaged blood vessels in zebrafish. They also indicate that ARPs are a major group of active constituents in Astragali Radix.

## Conclusion

In conclusion, this present study provides evidence supporting the hypothesis that P4, a polysaccharide fraction (50000 D < MW and DM < 0.1 μm) isolated from Astragali Radix partially restores chemical-induced blood vessel loss in the zebrafish model. Since polysaccharides isolated from natural products usually undergo the enzymatic breakdown of the sugar moiety in the cells of the gastrointestinal mucosa, or by enzymes secreted by the colon flora, to become active metabolites after oral consumption by humans, the study of the bioactivity of the polysaccharides required the development of an *in vivo *assay equipped with mammalian-equivalent drug metabolism systems. Recent studies suggest that drug screening in zebrafish shows a good correlation with clinical observations, and support its potential as a model for pharmacological assessment of small molecules, this is the first study to prove the concept of screening the bioactivity of polysaccharides in live zebrafish, whose drug metabolism systems were shown recently to have a high degree of functional similarity to that of mammals. Our findings also provide insight into the important role of polysaccharides in the effects of Astragali Radix for the treatment of various pathological conditions associated with deficient angiogenesis, such as ageing, stroke, ulcers and cardiovascular diseases.

## Abbreviations

Dpf: day(s) post fertilization; hpf: hour(s) post fertilization; D: Dalton; ARP: Astragali Radix polysaccharide; DMSO: Dimethyl Sulfoxide; VTKI (VRI): VEGFR tyrosine kinase inhibitor II; MW: Molecular Weight; Mn: number-average molecular weight; Mw: weight-average molecular weight; GPC: Gel Permeation Chromatography; GC: Gas Chromatography; DA: Dorsal Aorta; PCV: Posterior Cardinal Vein; DLAV: Longitudinal Anastamotic Vessel; ISV: Intersegmental Vessel; DM: Diameter.

## Competing interests

The authors declare that they have no competing interests.

## Authors' contributions

Guang Hu carried out experiments and drafted the manuscript. Gail B. Mahady revised the manuscript. Shang Li observed the experimental result and performed the statistical analysis. Maggie Pui Man Hoi participated in the design of the study. Simon Ming Yuen Lee and You-Hua Wang conceived of the study, and participated in its design and coordination. All authors read and approved the final manuscript.

**Figure 5:**
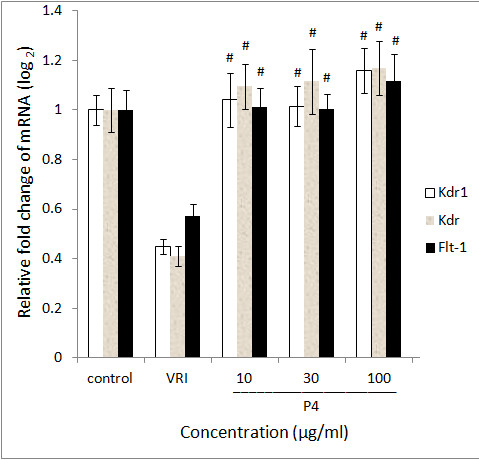
**Gene expression of P4 treated zebrafish**. Data are expressed as the mean ± SD, (n = 3), * *P *< 0.05, # *P *< 0.001 vs the VRI-only treatment group.

## Supplementary Material

Additional file 1 **GPC chromatogram of polysaccharides in P4**. A is the major polysaccharide peak in P4: retention time, 10.9 min; number-average molecular weight, 6.46 × 10^5^; Weight-average molecular weight, 1.61 × 10^6^; peak width, 2.49 min. Other peaks are monosaccharide peaks, solvent peak and other impurities. Click here for file

Additional file 2 **GC-MS chromatogram of acetylated-monosaccharide constituents in P4**. A (retention time 12.10 min) is the peak of internal standard (IS) inositol. B (retention time 12.68 min) is acetylated-glucuronic acid. C (retention time 12.77 min) is sorbitol hexaacetate. D (retention time 13.06 min) is galactose pentaacetate. Click here for file
