# Supplementary figures and images for: Polysaccharides from astragali radix restore chemical-induced blood vessel loss in zebrafish
Source: Vasc Cell. 2012 Feb 23;4:2. doi: 10.1186/2045-824X-4-2 (PMC3316134; doi:10.1186/2045-824X-4-2)

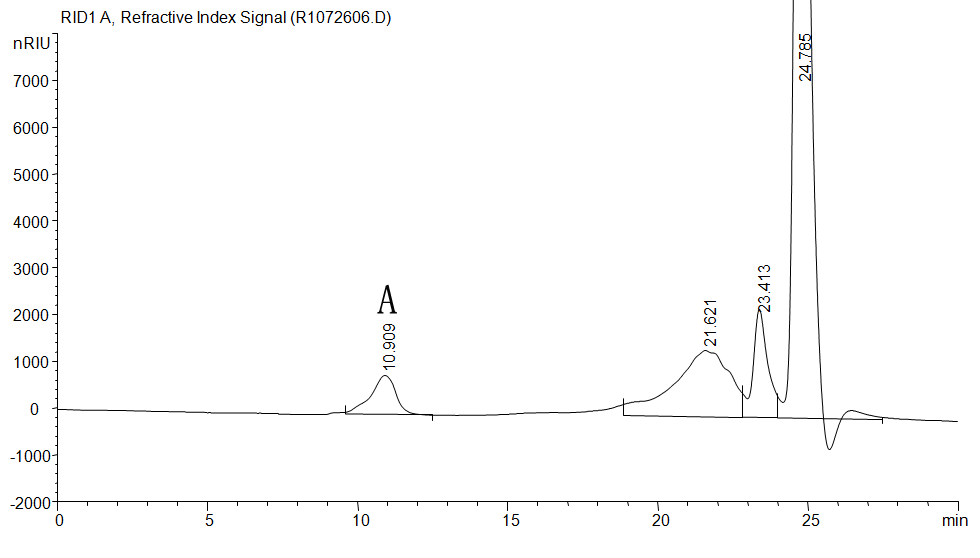

Supplement: Additional file 1 — GPC chromatogram of polysaccharides in P4. A is the major polysaccharide peak in P4: retention time, 10.9 min; number-average molecular weight, 6.46 × 10^5^; Weight-average molecular weight, 1.61 × 10^6^; peak width, 2.49 min. Other peaks are monosaccharide peaks, solvent peak and other impurities. [file 2045-824X-4-2-S1.TIFF]

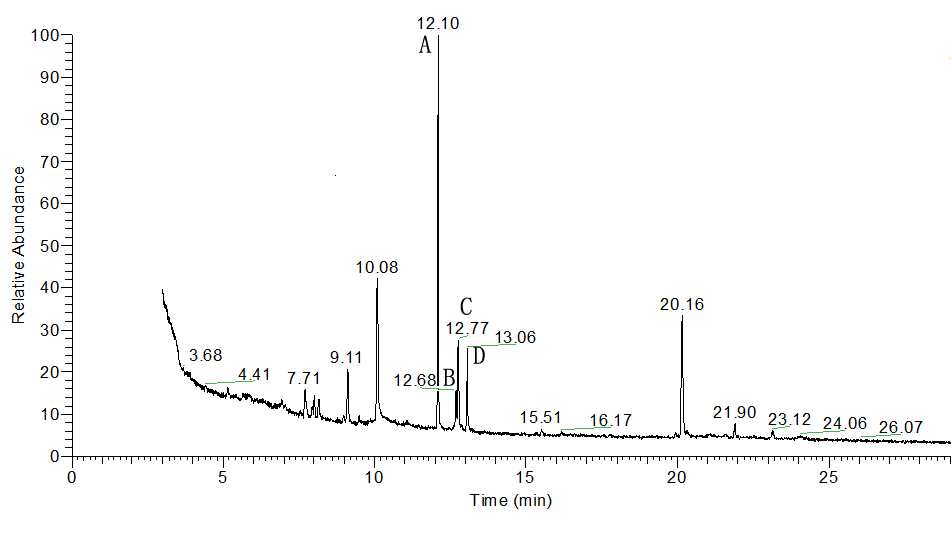

Supplement: Additional file 2 — GC-MS chromatogram of acetylated-monosaccharide constituents in P4. A (retention time 12.10 min) is the peak of internal standard (IS) inositol. B (retention time 12.68 min) is acetylated-glucuronic acid. C (retention time 12.77 min) is sorbitol hexaacetate. D (retention time 13.06 min) is galactose pentaacetate. [file 2045-824X-4-2-S2.TIFF]
